# Non-Convex Sparse and Low-Rank Based Robust Subspace Segmentation for Data Mining

**DOI:** 10.3390/s17071633

**Published:** 2017-07-15

**Authors:** Wenlong Cheng, Mingbo Zhao, Naixue Xiong, Kwok Tai Chui

**Affiliations:** 1 School of Information Science & Technology, Donghua University, Shanghai 200051, China; cheng.python@gmail.com; 2 Department of Electronic Engineering, City University of Hong Kong, Kowloon 999077, Hong Kong, China; ktchui3-c@my.cityu.edu.hk; 3 School of Mathematics and Computer Science, Northeastern State University, Tahlequah, OK 74464, USA; xiongnaixue@gmail.com

**Keywords:** subspace segmentation, low-rank representation, non-convex, LADMAP

## Abstract

Parsimony, including sparsity and low-rank, has shown great importance for data mining in social networks, particularly in tasks such as segmentation and recognition. Traditionally, such modeling approaches rely on an iterative algorithm that minimizes an objective function with convex *l*_1_-norm or nuclear norm constraints. However, the obtained results by convex optimization are usually suboptimal to solutions of original sparse or low-rank problems. In this paper, a novel robust subspace segmentation algorithm has been proposed by integrating *l_p_*-norm and Schatten *p*-norm constraints. Our so-obtained affinity graph can better capture local geometrical structure and the global information of the data. As a consequence, our algorithm is more generative, discriminative and robust. An efficient linearized alternating direction method is derived to realize our model. Extensive segmentation experiments are conducted on public datasets. The proposed algorithm is revealed to be more effective and robust compared to five existing algorithms.

## 1. Introduction

High dimensionality research for data mining is an essential topic in modern imaging applications, such as social networks and the Internet of Things (IoT). It is worth noting that data of high dimension is often supposed to reside in several subspaces of lower dimension. For instance, facial images with various lightning conditions and expressions lie in a union of a nine-dimensional linear subspace [1]. Moreover, moving motions in videos [2] and hand-written digits [3] can also be approximated by multiple low-dimensional subspaces. Inspiringly, these characteristics enable effective segmentation, recognition, and classification to be carried out. The problem of subspace segmentation [4] is formulated as determining the number of subspaces and partitions the data according to the intrinsic structure.

Many subspace segmentation algorithms have emerged in the past decades. Some of these methods are algebraic or statistical. Among the algebraic methods, generalized principal component analysis (GPCA) [5] is mostly widely used. GPCA characterizes the data subspace with the gradient of a polynomial, and segmentation is obtained by fitting the data with polynomials. However, the performance drops quickly in the presences of noise, and polynomial fitting computation is time consuming. As to the statistical algorithms, including random sample consensus (RANSAC) [6], factorization-based methods [7,8], and probabilistic principal component analysis (PPCA) [9], the estimation of exact subspace models exquisitely changed the performance of this type of methods. Recently, spectral-type methods [10] like sparse representation (SR) [7,11,12], low-rank representation (LRR) [13,14,15], and the extensions base on SR or LRR [16,17,18,19,20,21,22] have attracted much attention, as they are robust to noise [11,12,13,14], with strong theoretical backgrounds [16] and are easy to implement. Once an affinity graph is learned from SR or LRR, segmentation results can be obtained by means of spectral clustering. Therefore, building an affinity graph that accurately captures relevant data structures is a key point for SR- and LRR-based spectral-type models.

A good affinity graph should preserve the local geometrical structure, as well as the global information [11]. Recently, Elhamifar et al. [23] presented the sparse subspace clustering (SSC) based on an *l*_1_-graph. The *l*_1_-graph is constructed by using SR coefficients deduced in the *l*_1_-norm minimization to represent the relationships among samples. Attributed to SR, the *l*_1_-graph is sparse, capable of finding neighborhood according to the data and robust to noisy data. Inspired by *l*_1_-graph, various SR-graphs have been proposed [24]. Wang et al. [25] provided *l*_2_-Graph based subspace clustering to eliminate errors from various types of projection spaces. Peng et al. [26] proposed a unified framework for representation-based subspace clustering methods to cluster both the out-of-sample and the large-scale data. Peng et al. [27] introduced principal coefficients embedding to automatically identify the number of features, as well as learn the underlying subspace in the presence of Gaussian noise. Wang et al. [28] proposed a race lasso-based regularizer for multi-view data while keeping individual views well encapsulated. However, SR-based methods seek the sparsest representation coefficients of each data point individually, without a global structure regularizer. This drawback alters the robustness of these methods in the presence of outliers [15], when the data is not “clean” enough. To account for the underlying global information, Liu et al. [15] presented a two-step algorithm which firstly computes low-rank representation coefficients for data and then uses the coefficient matrix to build an affinity graph (LRR-graph). The LRR-graph jointly represents all the data by solving a nuclear norm optimization problem and, thus, becomes a better option at capturing global information. Numerous LRR-graph-based methods have also been proposed for spectral clustering. For example, Wang et al. [28] proposed a multi-graph Laplacian-regularized LRR to characterize the non-linear spectral graph structure from each view. Both SSC and LRR are based on convex relaxations of the initial problems, SSC uses *l*_1_-norm to approximate the number of non-zero elements, while LRR applies nuclear norm to compute the number of non-vanishing singular values. These convex relaxations yield solutions that deviate far from solutions to the original problems. Hence, it is desirable to rather use non-convex surrogates, without causing any significant increase in computational complexity.

A large number of studies on non-convex surrogates for *l*_0_-norm problem have been addressed recently. Xu et al. [29] introduced the *l*_1/2_-norm in noisy signal recovery with an efficient iterative half-thresholding algorithm. Similarly, Zhang et al. [30] proposed the *l_p_*-norm minimization with a generalized iterative shrinkage and thresholding method (GIST). Their study shows *l_p_*-norm based model is more effective on image denoising and image deburring. Numerous researchers also considered the other non-convex surrogate functions, such as homotopic *L*_0_-minimization [31], smoothly clipped absolute deviation (SCAD) [32], multi-stage convex relaxation [17], logarithm [33], half-quadratic regularization (HQR) [34], exponential-type penalty (ETP) [35], and minimax concave penalty (MCP) [36]. Recent years have also witnessed the progress in non-convex rank minimization functions. Mohan et al. [37] developed an efficient IRLS-*p* to minimize rank function, and improved the recovery performance for matrix completion. Xu et al. [18] introduced the *S*_1/2_-norm with an efficient ADMM solver for video background modeling. Kong et al. [38] proposed a Schatten *p*-norm constrained model to recover noisy data. Another popular non-convex rank minimization is the truncated nuclear norm [39]. They all compete with the state-of-the-art algorithms to some extent.

Combining the non-convex *l_p_*-norm regularizer with the Schatten *p*-norm (0 < *p* ≤ 1), in this study, we propose a robust method named non-convex sparse and low-rank-based robust subspace segmentation (*l_p_*S*p*SS). Our *l_p_*-norm error function can better predict errors, which further improves the robustness of subspace segmentation. Meanwhile, the Schatten *p*-norm-regularized objective function shows a better ability to approximate the rank of coefficient matrix compared with the nuclear norm. Our method can provide a more accurate description of the global information and better measurement of data redundancy. Thus, our new objective is to solve joint the *l_p_*-norm and Schatten *p*-norm (0 < *p* ≤ 1) minimization together. When *p*→0, our proposed *l_p_*S*p*SS turns to be more robust and effective than SR- and LRR-based subspace segmentation algorithms. In addition, we enforce non-negative constraint to the reconstruction coefficients, which aids interpretability and allows better solutions in numerous application areas such as text mining, computer vision, and bioinformatics. Traditionally, an alternating direction method (ADM) [40] can solve this optimization problem efficiently. However, to increase the speed and scalability of the algorithm, we choose an efficient solver commonly named the linearized alternating direction method with adaptive penalty (LADMAP) [19]. As it is based on fewer auxiliary parameters and without an inverse of its matrix, it is more efficient than ADM. Numerical experimental results verify our proposed method, which consistently obtains better segmentation results.

The rest of this paper is structured as follows: In Section 2, the notations, as well as the overview of SSC and LRR, will be presented. Section 3 is dedicated to introducing our novel non-convex sparse and low-rank based robust subspace segmentation. Section 4 conducts multiple numerical experiments to examine the effectiveness and robustness of *l_p_*S*p*SS. Section 5 concludes this work.

## 2. Background

This section is divided into three parts. First, the notation and definition are illustrated. The background of two algorithms, SSC and LRR, will be discussed in Section 2.2 and Section 2.3, respectively.

### 2.1. Notations and Definitions *∈*

Suppose $X=[x_{1},\cdot \cdot \cdot ,x_{N}]$ is an image matrix consists of *N* sufficiently dense data points ${\left\{x_{i}\in R^{m}\right\}}_{i=1}^{N}$, ${\left\{S_{i}\right\}}_{i=1}^{n}$ is an arrangement of *n* subspaces. Let {*x_i_*} be drawn from *n* subspaces $\left\{S_{1}\cup S_{2}\cdot \cdot \cdot \cup S_{n}\right\}$ of lower dimension. Given *X*, the goal of subspace segmentation is to partition the data points into the underlying low-dimensional subspaces.

The *l_p_*-norm (0 < *p* < ∞) of vector *x*∈*R^n×^*^1^ can be expressed as ${\left\|x\right\|}_{p}={\left({\sum_{1}^{n}\left\|x_{i}\right\|}^{p}\right)}^{1/p}$, in which *x_i_* is the *i*-th element. Therefore, the *p*-norm of *x*∈*R^n×^*^1^ to the power *p* can be expressed as ${\left\|x\right\|}_{p}^{p}={\sum_{1}^{n}\left\|x_{i}\right\|}^{p}$.

The Schatten *p*-norm of a matrix *x*∈*R^n×m^* is expressed as:

(1) $${\left\|X\right\|}_{S_{p}}={\left(\sum_{i=1}^{\min(m,n)}\sigma_{i}^{p}\right)}^{1/p}$$

in which 0 < *p* ≤ 1, and *σ*_i_ is the *i*-th largest singular value. Thus, it can be deduced that:

(2) $${\left\|X\right\|}_{S_{p}}^{p}=\left(\sum_{i=1}^{\min(m,n)}\sigma_{i}^{p}\right)$$

The Schatten 1-norm is just nuclear norm ${\left|X\right|}_{*}$, while the Schatten 0-norm is the approximation of the rank of *X*. Compared with ${\left|X\right|}_{*}$, ${\left|X\right|}_{S_{p}}$ is a better approximation of the rank of *X*.

### 2.2. Sparse Subspace Clustering

Recently, SSC [16] has grabbed considerable attention. The hypothesis states that data are drawn from several subspaces of lower dimension, and can be sparsely self-expressed. More formally, SSC aims to solve the following program:

(3) $$\begin{array}{ccc} \underset{Z}{\min}{\sum_{i}\left\|Z_{i}\right\|}_{0}s.t.X=XZ & and & diag(Z)=0 \end{array}$$

where $Z_{i}={\left[z_{i1},z_{i2},\cdot \cdot \cdot ,z_{iN}\right]}^{T}\in R^{N}$ are the reconstruction coefficients, and ${\left\|z\right\|}_{0}$ refers to the number of nonzero values. As it is difficult to solve this non-convex objective, a convex *l*_1_ minimization problem is proposed by solving the following program:

(4) $$\begin{array}{ccc} \underset{Z}{\min}{\left\|Z\right\|}_{1}s.t.X=XZ & and & diag(Z)=0 \end{array}$$

The minimization problem in Equation (4) can be concluded using the alternating direction method of multipliers (ADMM) [19]. Afterwards, the coefficient matrix Z can be utilized to construct the affinity matrix as $W=\left|Z\right|+\left|Z^{T}\right|$. Finally, *W* is performed via spectral clustering and the segmentation result is drawn. While SSC works well in practice, the model is invalid when the obtained similarity graph is poorly connected (we refer readers to Soltanolkotabi et al. [41] for very recent results in this direction).

### 2.3. Low-Rank Representation-Based Subspace Segmentation

The difference between LRR and SSC is that, LRR seeks the lowest rank representation *Z* but not the sparsest representation. LRR is based on the assumption that for observed data $X\in R^{d\times N}$ drawn from *n* low-dimensional subspaces, and the rank of coefficient matrix $r=rank(Z)=\sum_{i=1}^{n}z_{i}$ is assumed to be much smaller than min{d,N}. The LRR is formulated as:

(5) $$\begin{array}{cc} \underset{z}{\min}\;rank(Z) & s.t.X=XZ \end{array}$$

As the rank function minimization is non-convex, Equation (5) can be reformulated as the following convex minimization problem:

(6) $$\begin{array}{cc} \underset{z}{\min}{\left\|Z\right\|}_{*} & s.t.X=XZ \end{array}$$

in which ${\left\|Z\right\|}_{*}$ is the nuclear norm, which yields a good approximation to the matrix rank of *Z*. Singular value threshold (SVD) can be used to efficiently solve Equation (6), when there is no error present in *X*.

When the data *X* is noisy, an extension of LRR is proposed as follows:

(7) $$\begin{array}{cc} \underset{Z,E}{\min}{\left\|Z\right\|}_{*}+\lambda E_{p} & s.t.X=XZ+E \end{array}$$

in which *λ* ≥ 0 is the tradeoff parameter, trading off low rankness between reconstruction error. $E_{p}\in R^{d\times N}$ is the noise term with different regularization strategies, which depends on the property of *E_p_*. When the noise term is Gaussian noise, ${\left\|E\right\|}_{p}={\left\|E\right\|}_{F}^{2}$, in which ${\left\|\cdot \right\|}_{F}$ refers to the Frobenius norm. When the noise term are entry-wise corruptions, ${\left\|E\right\|}_{p}={\left\|E\right\|}_{1}$, in which ${\left\|\cdot \right\|}_{1}$ refers to the *l*_1_ norm. When the noise term are sample-specific corruption and outliers, ${\left\|E\right\|}_{p}={\left\|E\right\|}_{2,1}$, in which ${\left\|E\right\|}_{2,1}=\sum_{i=1}^{N}\sqrt{\sum_{i=1}^{d}E_{ij}^{2}}=\sum_{i=1}^{N}{\left\|E_{j}\right\|}_{2}$ refers to the *l*_2_*_,_*_1_ norm. Equation (7) can be solved by ADMM [19] to obtain the coefficient matrix *Z*. Afterwards, the coefficient matrix Z can be utilized for the construction of affinity matrix $W=\left|Z\right|+\left|Z^{T}\right|$. Finally, spectral clustering can be applied to *W* for segmentation results.

## 3. Non-Convex Sparse and Low-Rank Based Robust Subspace Segmentation

In this section, we first propose the non-convex sparse and low-rank-ased robust subspace segmentation model, in which we combine the *l_p_*-norm with the Schatten *p*-norm together for clustering, and then use LADMAP to solve *l_p_*S*_p_*SS. Finally, we analyze of the time complexity of *l_p_*S*p*SS.

### 3.1. Model of l_p_SpSS

We consider the non-convex sparse and low-rank-based subspace segmentation for data contaminated by noise and corruption. Notice that the nuclear norm is replaced by the Schatten *p*-norm, when *p* is smaller than 1, the underlying global information can be captured more effectively. Additionally, the *l_p_* norm (0 < *p* ≤ 1) of the coefficient matrix is also introduced as an error function, in order to harvest stronger robustness to noise [42]. It has been demonstrated in some recent research [43] that the Schatten *p*-norm is more powerful than the nuclear norm in matrix completion, and the recovery performance of the *l_p_*-norm is also superior to the convex *l*_1_-norm [36]. Our *l_p_*S*pSS* will surely be more effective than the convex methods.

We begin by considering the relaxed low-rank subspace segmentation problem, which is equivalent to:

(8) $$\begin{array}{l} \underset{Z,E}{\min}{\left\|Z\right\|}_{S_{p}}^{p}+\beta {\left\|Z\right\|}_{p}^{p}+\lambda {\left\|E\right\|}_{2,1}\\ s.t.\;X=XZ+E,Z\geq 0 \end{array}$$

In which, the first term ${\left\|Z\right\|}_{p}^{p}$ is the *l_p_*-norm, which improves the integration of the local geometrical structure. Meanwhile, the second term ${\left\|Z\right\|}_{S_{p}}^{p}$ is the Schatten *p*-norm, which can better approximate the rank of *Z*. Moreover, the third term reconstruction error ${\left\|E\right\|}_{2,1}$ is the *l*_2,1_ norm, which can better characterize errors like corruption and outliers. *β* and *λ* are trade-off parameters. Regarding the widely-used non-negative constraint (*Z* ≥ 0), which is to ensure direct use of the reconstruction coefficients in the affinity graph construction.

### 3.2. Solution to l_p_SpSS

#### 3.2.1. Brief Description of LADMAP

We adopt LADMAP [19] to solve the objective function (Equation (8)) constrained by the *l_p_*-norm norm and Schatten-*p* regularizers. An auxiliary variable *W* is introduced and the optimization problem becomes separable. Thus, Equation (8) is rewritten as:

(9) $$\begin{array}{l} \underset{Z,E}{\min}{\left\|Z\right\|}_{S_{p}}^{p}+\beta {\left\|Z\right\|}_{p}^{p}+\lambda {\left\|E\right\|}_{2,1}\\ s.t.\;X=XZ+E,Z=W,W\geq 0 \end{array}$$

To remove two linear constraints in Equation (9), we introduce two Lagrange multipliers *Y*_1_ and *Y*_2_, hence, the optimization problem is defined using the following Lagrangian function:

(10) $$\begin{array}{cc} L(Z,W,E,Y_{1},Y_{2},u) & ={\left\|Z\right\|}_{S_{p}}^{p}+\beta {\left\|W\right\|}_{p}^{p}+\lambda {\left\|E\right\|}_{2,1}+\langle Y_{1},X-XZ-E\rangle +\langle Y_{2},Z-W\rangle +\frac{\mu }{2}({\left\|X-XZ-E\right\|}_{F}^{2})+({\left\|Z-W\right\|}_{F}^{2})\\ & ={\left\|Z\right\|}_{S_{p}}^{p}+\beta {\left\|W\right\|}_{p}^{p}+\lambda {\left\|E\right\|}_{2,1}+q(Z,W,E,Y_{1},Y_{2},\mu )-\frac{1}{2\mu }({\left\|Y_{1}\right\|}_{F}^{2}+{\left\|Y_{2}\right\|}_{F}^{2}) \end{array}$$

where $q\left(Z,W,E,Y_{1},Y_{2},\mu \right)=\frac{\mu }{2}\left({\left\|X-XZ-E+Y_{1}/\mu \right\|}_{F}^{2}+{\left\|Z-W+Y_{2}/\mu \right\|}_{F}^{2}\right)$, *Y*_1_ and *Y*_2_ are the Lagrange multipliers, and *μ* ≥ 0 is a trade-off parameter. We solve Equation (10) by minimizing *L* to update each variable with the other variables fixed. The updating schemes at each iteration can be designed as follows:

(11) $$W(k+1)=\arg\underset{W\geq 0}{\min}\beta {\left\|W\right\|}_{p}^{p}+\frac{\mu (k)}{2}{\left\|Z(k+1)-W+Y_{2}(k)/\mu (k)\right\|}_{F}^{2}$$

(12) $$\begin{array}{lll} Z(k+1) & = & \arg\underset{Z}{\min}{\left\|Z\right\|}_{S_{p}}^{p}+\langle \nabla_{Z}q(Z(k),W(k),E(k),Y_{1}(k),Y_{2}(k),\mu (k),Z-Z(k)\rangle +\frac{\mu (k)\theta }{2}{\left\|Z-Z(k)\right\|}_{F}^{2}\\ & = & \arg\underset{Z}{\min}{\left\|Z\right\|}_{S_{p}}^{P}+\;\\ & & \frac{\theta \mu (k)}{2}{\left|Z-Z(k)+[-X^{T}(X-XZ(k)-E(k)+\frac{Y_{1}(k)}{\mu (k)})+(Z(k)-W(k)+\frac{Y_{2}(k)}{\mu (k)})]/\theta \right|}_{F}^{2} \end{array}$$

(13) $$E(k+1)=\arg\underset{E}{\min}\lambda {\left\|E\right\|}_{2,1}+\frac{u(k)}{2}{\left\|X-XZ(k+1)+\frac{Y_{1}(k)}{u(k)}-E\right\|}_{F}^{2}=\Omega_{\lambda u{\left(k\right)}^{-1}}(X-XZ(k+1)+\frac{Y_{1}(k)}{\mu (k)})$$

In Equation (12), $\nabla_{Z}q$ is the partial differential of *q* with respect to *Z*, $\theta ={\left\|X\right\|}_{F}^{2}$. In particular, the detailed procedures of LADMAP are shown in Algorithm 1. The first, Equation (11), and the second, Equation (12), are solved using the following subsections. The last convex problem (Equation (13)) can solved by the *l*_2,1_-norm minimization operator [15].

**Algorithm 1.** LADMAP for solving Equation (9).**Input:** Data *X*, tradeoff variables *β* > 0, *λ* > 0**Initialize: *Z***(0) = ***W***(0) = ***E***(0), ***Y*_1_**(0) = ***Y*_1_** (0) = 0, *µ*(0) = 0.1, *µ_max_* = 10^7^, *ρ*_0_ = 1.1, *ε*_1_ = 10^−^^7^, *ε*_2_ = 10^−^^6^, *k* = 0,$\theta ={\left\|X\right\|}_{F}^{2}$, and the number of maximum iteration *MaxIter* = 1000.**While** not converged and *k ≤ MaxIter*
**do**
(1)Compute *W*(*k*+1) by solving Equation (11) with *Z*(*k*+1)*, E*(*k*+1) are fixed;(2)Compute *Z*(*k*+1) by solving Equation (12) with *W*(*k*+1)*, E*(*k*+1) are fixed;(3)Compute *E*(*k*+1) by solving Equation (13) with *W*(*k*+1)*, Z*(*k*+1) are fixed;(4)Compute the multipliers ***Y*_1_** (*k* + 1)and ***Y*_2_** (*k* + 1) as follows:$\{\begin{array}{c} Y_{1}(k+1)=Y_{1}(k)+\mu (k)(X-XZ(k+1)-E(k+1))\\ Y_{2}(k+1)=Y_{2}(k)+\mu (k)(Z(k+1)-W(k+1)) \end{array}$(5)Compute *µ*(*k* + 1) as follows:$\mu (k+1)=\min(\mu_{\max},\rho \mu (k))$, in which$\rho =\{\begin{array}{c} \rho_{0},if\mu (k)\Omega /{\left\|X\right\|}_{F}\leq \varepsilon_{2}\\ 1,otherwise \end{array}$(6)Converges when$\{\begin{array}{c} {\left\|X-XZ(k)-E(k)\right\|}_{F}/{\left\|X\right\|}_{F}\leq \varepsilon_{1}or\\ \mu (k)\Omega /{\left\|X\right\|}_{F}\leq \varepsilon_{2} \end{array}$In which $\Omega =\max(\sqrt{\theta }{\left\|Z(k)-Z(k+1)\right\|}_{F},{\left\|W(k)-W(k+1)\right\|}_{F},{\left\|E(k)-E(k+1)\right\|}_{F})$(7)Increment of iteration: *k* = *k* + 1. **End while** **Output**: The optimal solution ***W***, ***Z*** and ***E***.

#### 3.2.2. Solving the Non-Convex *l_p_*-Norm Minimization Subproblem (Equation (11))

For each element in $\left\{X_{i}{}_{j}|\left(i,\;j\right)\in \Omega \right\}$, we can decouple Equation (11) into a simplified formula:

(14) $$\min\frac{1}{2}{\left(x-y\right)}^{2}+\lambda {\left|x\right|}^{p}$$

Recently, Zhang et al. solved this *l_p_-*norm optimization problem via the proposed GIST [30]. For *l_p_-*norm minimization, the thresholding function is:

(15) $$\tau_{p}(\lambda )={\left(2\lambda (1-p)\right)}^{1/(2-p)}+\lambda p{\left(2\lambda (1-p)\right)}^{\left(p-1)/(2-p\right)}$$

Meanwhile, the generalized soft-thresholding function is:

(16) $$T_{p}(y;\lambda )=\{\begin{array}{cc} 0, & if\left|y\right|\leq \tau_{p}(\lambda )\\ \mathrm{sgn}(y)S_{p}(\left|y\right|;\lambda ), & if\left|y\right|>\tau_{p}(\lambda ) \end{array}$$

The corresponding thresholding rule in the generalized soft-thresholding function is $T_{p}^{GST}(y;\lambda )=0$ when $\left|y\right|\leq \tau_{p}^{GST}(\lambda )$, and corresponding shrinkage rule is $T_{p}^{GST}(y;\lambda )=\mathrm{sgn}(y)S_{p}^{GST}(\left|y\right|;\lambda )$ when $\left|y\right|>\tau_{p}^{GST}(\lambda )$.

#### 3.2.3. Solving the Non-Convex Schatten *p*-Norm Minimization Subproblem (Equation (12))

We can reformulate Equation (12) as the following simplified notation:

(17) $$\underset{X}{\min}\frac{1}{2}{\left\|X-A\right\|}_{F}^{2}+\lambda X_{s_{p}}^{p}$$

After applying SVD on *X*, *X* is decomposed into summation of *r* rank-*p* matrices *X = UΔV^T^*. Here, *U* is the left singular vector, *Δ* is the non-zero singular diagonal matrix, and *V* is the right singular vector. The *i*-th singular value *δ_i_* is solved by:

(18) $$\underset{\delta_{i}\geq 0}{\min}\frac{1}{2}{\left(\delta_{i}-\sigma_{i}\right)}^{2}+\lambda \delta_{i}^{p}$$

Equation (16) can be used to solve Equation (18) again. For *p* = 1, we can obtain the same solution with nuclear norm minimization [44].

### 3.3. Convergence and Computational Complexity Analysis

Although Algorithm 1 is described in three major alternative steps for solving *W*, *Z,* and *E*, we can actually combine steps for *Z* and *E* easily into one larger block step by simultaneously solving for (*Z, E*). Thus, the convergence conclusion of two variables LADMAP in [45] can be applied to our case. Finally, the convergence of the algorithm is ensured.

Suppose the size of *X* are *d ×* *n, k* is number of total iterations, and *r* is the lowest rank for *X*. The major time consumption of Algorithm 1 is mainly determined by Step 2, as it involves time-consuming SVDs. In Step 1, each component of $\nabla_{Z_{k}}q$ can be computed in *O*(*rn*^2^) by using the skinny SVD to update *W*. In Step 2, the complexity of the SVD to update *Z* is approximately *O*(*d*^2^*n*). In Step 3, the computation complexity of *l*_2,1_ minimization operator is about *O*(*dn*). The total complexity is, thus, *O*(*krn*^2^ + *kd*^2^*n*). Since *r* ≤ *min*(*d, n*), the time cost is, at most, *O*(*krn*^2^)*.*

### 3.4. Affinity Graph for Subspace Segmentation

Once Equation (9) was solved by LADMAP, we can obtain the optimal coefficient matrix Z^*^. Since every sample is reconstructed by its neighbors, Z* naturally characterizes the relationships among samples. Such information is a good indicator of similarity among samples, we use the reconstruction coefficients to build the affinity graph. The non-convex *l_p_*-norm ensures that each sample only connects to few samples. As a result, the weights of the graph tend to be sparse. While the non-convex Schatten *p*-norm ensures samples lying in the same subspace are highly correlated and tend to be assigned into the same cluster, Z* is theoretically able to capture the global information, and the graph weights are constrained with non-negativity, as they reflect similarities between data points.

After obtaining the coefficient matrix Z^*^, the reconstruction coefficients of each sample are normalized and thresholded to zero. Therefore, the obtained normalized sparse ${\hat{Z}}^{*}$ can be used to compute the affinity graph $W=({\hat{Z}}^{*}+{\left({\hat{Z}}^{*}\right)}^{T})/2$. Finally, *W* carries out spectral clustering to obtain the segmentation results. Our proposed non-convex sparse and low-rank based subspace segmentation is outlined in Algorithm 2.

**Algorithm 2.**
*l_p_*S*p*SS.**Input:** Matrix $X=[x_{1},x_{2},\cdot \cdot \cdot ,x_{n}]\in R^{d\times n}$, tradeoff parameters *β*, *λ*
Normalize each sample *x_i_* to obtain $\hat{X}=[{\hat{x}}_{1},{\hat{x}}_{2},\cdot \cdot \cdot ,{\hat{x}}_{n}]$. Solve the non-convex sparse and low-rank constrained program by Algorithm 1:$\begin{array}{l} \min{\left\|Z\right\|}_{S_{p}}^{p}+\beta {\left\|Z\right\|}_{p}^{p}+\lambda {\left\|E\right\|}_{2}^{p},\\ s.t.X=XZ+E,Z\geq 0 \end{array}$and get (Z*, E*). Normalize coefficient matrix$Z^{*}$, and threshold small values by *θ* to obtain ${\hat{Z}}^{*}$.Set weights for affinity graph by $W=({\hat{Z}}^{*}+({\left({\hat{Z}}^{*}\right)}^{T})/2$.The data is segmented by spectral clustering.
**Output:** The segmentation results.

## 4. Experimental Evaluation and Discussion

In the following, we will discuss the performance of our proposed *l_p_*S*p*SS model. Firstly, the experimental setting is detailed in Section 4.1. From Section 4.2，Section 4.3，Section 4.4, we will test the segmentation performance of *l_p_*S*p*SS on CMU-PIE, COIL20, and USPS. In Section 4.5, we will examine the robustness of *l_p_*S*p*SS to block occlusions and pixel corruptions. Finally, the discussion of experimental results will be given in Section 4.6.

### 4.1. Experimental Settings

The proposed *l_p_*S*p*SS approach will be evaluated on realistic images and compared with five related works. We use four publicly-available datasets, including CMU-PIE [46], COIL20 [47], USPS [48], and Extended Yale B [49]. Among them, datasets [46] and [49] contain face images with various poses/illuminations/facial expressions, COIL20 consists of different general objects, and USPS includes handwritten digit images. Our proposed *l_p_*S*p*SS will be compared with five segmentation methods, including PCA, SSC [16], LRR [15], and NNLRS [50], while K-means serves as a baseline for comparison.

We adopt the same experimental settings as Zhuang’s work [50]. For the compared methods, a grid search strategy is used for selecting model parameters, and the optimal segmentation is achieved by tuning the parameters carefully. As to our *l_p_*S*p*SS, there are two regularized parameters, *β* and *λ,* affecting its performance. We take a stepwise selection strategy to search the best parameters. For example, we search the possible candidate interval *λ* may exit, with *β* fixed, and alternatively search *λ’s* most possible candidate interval, with *β* fixed. Finally, the best values are found in a two-dimensional candidate space of (*β, λ*).

To quantitatively and effectively measure the segmentation performance, two quantity metrics, namely accuracy (AC) and normalized mutual information (NMI) [51], are used in our experiments. All the experiments are implemented by MATLAB, on a MacBook Pro with a 2.6 GHz Intel Core i7 CPU and 16 GB memory.

### 4.2. Segmentation Results on CMU-PIE Database

**Figure 1:**
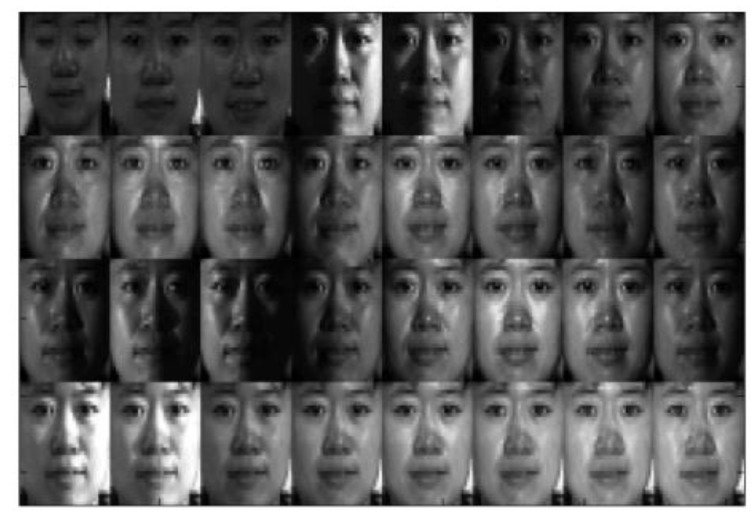
Typical examples of the CMU-PIE dataset.

In this experiment, we compare *l_p_*S*p*SS with the other five methods on the CMU-PIE facial images dataset. It includes 41,368 pictures of 68 persons, acquired with various postures and lighting scenarios. The resolution of each image is 32 × 32 = 1024 pixels. Typical examples of CMU-PIE are shown in Figure 1. For each given cluster number *K* = 4*,...,* 68 in the whole dataset, the segmentation results with different *K* were averaged on the twenty tests. The averaged segmentation performance of proposed and existing algorithms on the CMU-PIE dataset [46] are reported in Table 1.

We can see that our proposed *l_p_*S*p*SS achieves the best segmentation AC and NMI on CMU-PIE dataset, which proves the effectiveness of our *l_p_*S*p*SS. For example, the average segmentation accuracy of NNLRS and *l_p_*S*p*SS are 84.1% and 89.8%, respectively. *l_p_*S*p*SS improves the segmentation accuracy by 5.7% compared with NNLRS (the second best algorithm). The improvement of *l_p_*S*p*SS indicates the importance of the non-convex SR and LRR affinity graph.

### 4.3. Segmentation Results on COIL20 Database

**Figure 2:**
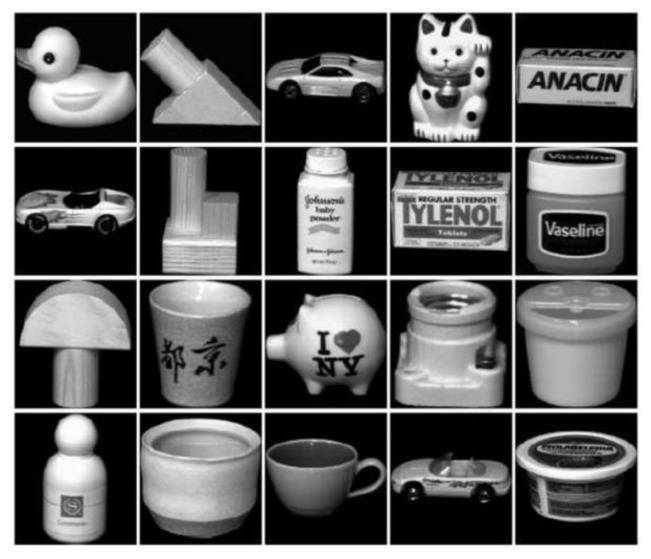
Typical examples of the COIL20 dataset.

When it comes to the evaluation using second dataset COIL20 [49], the proposed *l_p_*S*p*SS is compared with five existing algorithms. This dataset contains 1440 images of 20 objects, with 72 different views. The resolution of each picture is 32 × 32 = 1024 pixels. Typical examples of COIL20 are shown in Figure 2. For each given cluster number *K* = 2*,...,*20 in the whole dataset, the segmentation results with different *K* were averaged on the twenty tests. The averaged segmentation performances of the proposed and existing algorithms on the COIL20 dataset [47] are reported in Table 2.

Experimental results on COIL20 indicates that our *l_p_*S*p*SS outperforms the other five existing algorithms. For example, the average AC and NMI of *l_p_*S*p*SS are 90.2% and 91.0%, which are higher than for NNLRS (the second best algorithm) by 2.0% and 1.5%, respectively. Especially, when the cluster number is large, the superiority of *l_p_*S*p*SS is very obvious.

### 4.4. Segmentation Results on USPS Handwritten Digit Dataset

**Figure 3:**
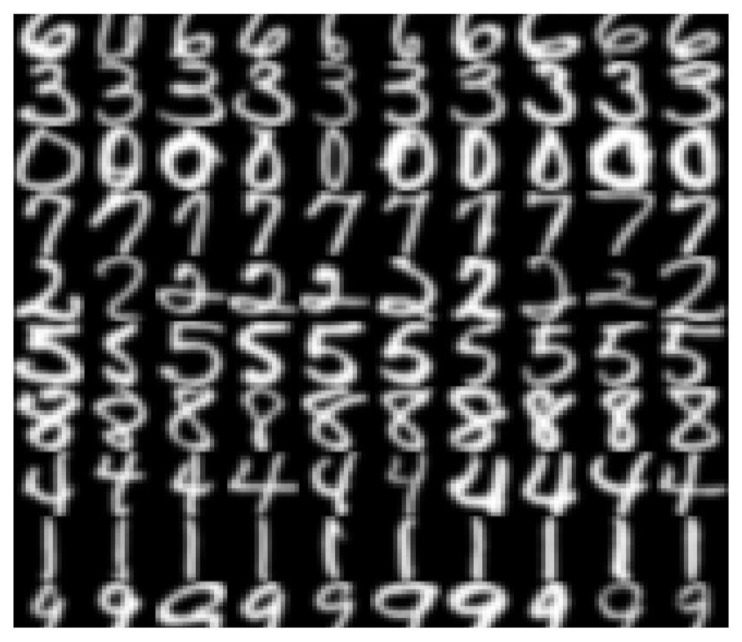
Typical examples of USPS dataset.

In this experiment, we compare *l_p_*S*p*SS with the other five methods on the USPS handwritten digit dataset. It contains 9298 images of 10 classes, with a variety of orientations. The resolution of each picture is 16 × 16 = 256 pixels. Figure 3 shows typical sample images in the USPS dataset. For each given cluster number *K* = 2,*...*,10, the segmentation results with different *K* were averaged on the twenty tests. The averaged segmentation performances of proposed and existing algorithms on the USPS handwritten digit dataset are reported in Table 3.

Table 3 shows that our proposed *l_p_*S*p*SS still obtains the best segmentation performance. This result demonstrates that a non-convex sparse and low-rank graph is better to model complex related data than traditional SR and LRR based graphs. Experimental results have demonstrated that our proposed *l_p_*S*p*SS model can not only represent the global information, but also preserves the local geometrical structures in the data by incorporating the non-convex *l_p_*-norm regularizer.

### 4.5. Segmentation Results on Dataset with Block Occlusions and Pixel Corruptions

**Figure 4:**
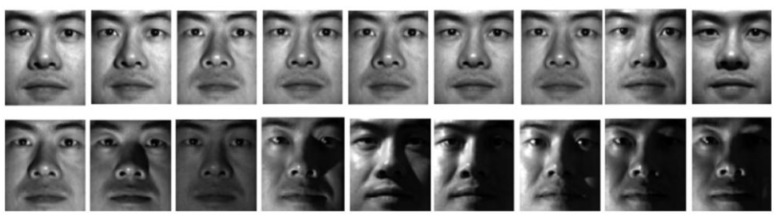
Typical sample images of Extended Yale B dataset.

Finally, we evaluate the robustness of each model on the more challenging Extended Yale B face dataset. It has 38 × 64 facial images, with various lighting scenarios. To reduce the resources and budget, the resolution of each picture is downsized to 96 × 84. This dataset more challenging for subspace segmentation, as 50% of the samples with hard shadows or specularities. Figure 4 shows typical sample images of Extended Yale B.

**Figure 5:**
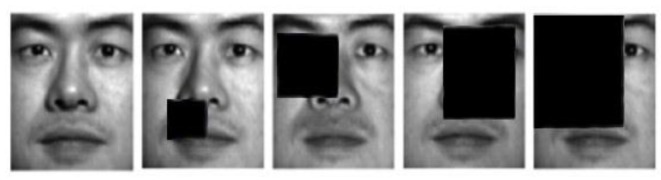
Typical sample images of the corrupted dataset.

We select 1134 images from first 18 individuals to evaluate the different methods. Two types of corruptions are introduced into this experiment. For Type I block occlusions, different block sizes (from 5 × 5 to 20 × 20) are added to randomly selected locations of the images. For Type II random pixel corruption, randomly-chosen pixels on each images are substituted with equally-distributed random values. The proportion of corrupted pixels per image is from 0 to 20%. Some examples from corrupted Extended Yale B face images are shown in Figure 5. The averaged segmentation AC of proposed and existing algorithms with multiple block occlusions and pixel corruptions are tabulated in Table 4 as well as Table 5.

Both experimental results show that our *l_p_*S*p*SS achieves the best segmentation results again. Segmentation results suggest that proposed *l_p_*S*p*SS is more robust than compared methods, especially when a significant portion of the realistic samples are corrupted.

### 4.6. Discussions

Our *l_p_*S*p*SS outperforms five existing subspace segmentation algorithms. Especially, in the case of CMU-PIE face dataset, the improvement by *l_p_*S*p*SS is largest. Our affinity graph can capture the local geometrical structure, as well as the global information of the data, hence, is both generative and discriminative.

*l_p_*S*p*SS is more robust than the other compared methods, which can properly deal with multiple noises. Images in Extended Yale B dataset contain different errors, including block occlusions, pixel corruptions, illuminations, partition them is challenging. However, *l_p_*-norm and Schatten *p*-norm are introduced for *l_p_*S*p*SS affinity graph construction. Therefore, our model can better predict errors and is a better measurement for data redundancy.

The segmentation performance of SSC and LRR are almost the same. For example, the segmentation accuracy of SSC on CMU-PIE is 0.9% better than LRR, while the performance of LRR on USPS is 0.7% higher than SSC. The segmentation results heavily depends on the intrinsic structure of the testing dataset, it is difficult to determine which one is better.

The LRR-based algorithm is robust in handling noisy data. It aims at obtaining the low rankness of coefficient matrix, thus, the LRR-based methods can better model the global information. Furthermore, LRR can find similar clusters which measure the data redundancy, ensuring high quality and stability of the segmentation results. For data heavily contaminated with corruptions or outliers, the model can find lower ranks that will be more robust to noise.

However, SVD computation for Schatten *p*-norm minimization is performed in each iteration, which is very time consuming, and the best segmentation results are not achieved at the lowest value of *p*. Hence, we will be focused on the study of speeding up the Schatten *p*-norm solver and the selection of best *p* values in our future work.

## 5. Conclusions

This paper presents an accurate and robust for subspace segmentation, named *l_p_*S*p*SS, by introducing the non-convex *l_p_*-norm and Schatten *p*-norm minimization. Taking advantages from the original sparsity and low rankness of data of high dimension, both local geometrical structure and the global information of the data can be learnt. A linearized alternating direction method with adaptive penalty (LADMAP) is also introduced to search for optimal solutions. Numerous experiments on CMU-PIE, COIL20, USPS, and Extended Yale B verify the effectiveness and robustness of our *l_p_*S*p*SS compared to five existing works.

## Figures and Tables

**Table 1:** Segmentation results of proposed and existing algorithms on the CMU-PIE dataset.

Clusters *K*	AC (%)						NMI (%)					
	K-Means	PCA	SSC	LRR	NNLRS	*l_p_*S*p*SS	K-Means	PCA	SSC	LRR	NNLRS	*l_p_*S*p*SS
4	48.5	52.4	100	100	100	100	64.1	66.8	100	100	100	100
12	41.9	47.3	81.5	89.5	79	91.1	63.4	62.9	84.6	95.5	96.7	96.8
20	38.8	36.7	80.6	81.3	88.3	92.2	62.3	58.8	85.9	90.8	94.3	98.5
28	35.7	34.9	78.2	77.4	87.9	91.9	61.7	61.1	86.4	89.9	94	96.9
36	34.3	34.7	77.1	68.8	78.7	87.6	60.5	60.6	85.7	82.3	93.6	96.5
44	33.8	33.7	75.1	71.7	81.3	84.2	59.1	62.6	84.7	84.2	93.8	95.7
52	33.1	33.7	69.9	71.1	75.4	88.2	58.1	61.6	85.3	84.9	93.1	96
60	33	33.2	68.1	65.6	79.6	84.7	52.7	53.6	84.9	80.2	93.5	95.4
68	31	32.8	66.7	65.1	86.2	88.1	46.8	46.7	85.5	79.3	87.9	96.6
Average	36.7	37.7	74.7	73.8	84.1	89.8	58.7	59.4	85.4	87.4	93.4	96.9

**Table 2:** Segmentation results of proposed and existing algorithms on the COIL20 dataset.

Clusters *K*	AC (%)						NMI (%)					
	K-means	PCA	SSC	LRR	NNLRS	*l_p_*S*p*SS	K-Means	PCA	SSC	LRR	NNLRS	*l_p_*S*p*SS
2	88.3	88.1	96.2	90.3	98.3	98.7	80.4	81.4	88.7	90.8	92.2	92.6
4	84.7	84	81.5	88.8	96.1	98	77.5	78.7	84.6	87.3	87.7	91.2
6	74.5	83.4	80.6	83.9	94.6	95.1	72.3	73.8	85.9	86.2	89.1	90.6
8	73.8	71.1	78.2	77	86.9	92.5	75.3	74.8	86.4	86.8	90.1	88.5
10	71.2	69.4	77.1	74.9	87.2	89.1	74.1	74.8	85.7	85.9	89.5	91.9
12	68.8	68.5	75.1	70.3	86.8	87.9	75.4	75.6	84.7	86.8	90	89.5
14	65.2	66.3	69.9	66.5	84.9	86.6	74.1	75	85.3	85.6	88.6	92.1
16	66.4	67.3	68.1	67	85.5	87.7	74.8	74.6	84.9	84.1	88.9	91.7
18	63.5	65.8	67.1	65.8	83.1	84.6	74.9	74.7	84.4	84.6	88.8	91.1
20	62.8	64.3	66.7	64	78.8	81.7	75.8	74.1	85.5	86.4	90.1	91
Average	71.9	72.8	76.1	74.9	88.2	90.2	75.5	75.8	85.6	86.5	89.5	91

**Table 3:** Segmentation results of proposed and existing algorithms USPS handwritten digit dataset.

Clusters *K*	AC (%)						NMI (%)					
	K-Means	PCA	SSC	LRR	NNLRS	*l_p_*S*p*SS	K-Means	PCA	SSC	LRR	NNLRS	*l_p_*S*p*SS
2	94.1	94.3	94.2	94.6	96.6	98.3	71.9	72.2	81.4	73.8	79.3	82.9
3	88.1	88.8	89.3	89.3	94.7	96.4	71.1	71.4	79.8	75.9	80.3	83.4
4	82.2	79.2	83.3	84	90.1	91.2	67.1	68.2	79.4	72.3	77.3	80.3
5	79.1	78.2	79.1	80.8	87.3	88.3	65	66.7	77.9	70.6	79.2	81.1
6	77.4	74.3	75.2	75.1	88.3	90.2	65.1	66.7	76.3	73.6	75.7	79.1
7	74.8	73.3	74.2	75.6	82.7	84.3	62.7	63.2	81.4	69.8	74.5	75
8	71.5	71.8	74.3	76.3	80.6	82.4	61.3	63.4	79.8	68.9	73.2	75.1
9	68.7	69.2	75.3	75	79.4	80.6	59.9	60.2	79.4	67.3	72.7	74.7
10	65.4	63.3	74.2	74.3	75.1	77.4	59.4	60.7	77.3	66.6	71.6	72.6
Average	77.9	76.9	79.9	80.6	86.1	87.7	64.8	65.9	79.2	71	76	78.2

**Table 4:** Segmentation results of proposed and existing algorithms on Extended Yale B dataset with multiple block occlusions.

Block Size	AC (%)						NMI (%)					
	K-Means	PCA	SSC	LRR	NNLRS	*l_p_*S*p*SS	K-Means	PCA	SSC	LRR	NNLRS	*l_p_*S*p*SS
5 × 5	13.6	15.1	78.5	88.3	89.5	90.6	14.2	16.1	79.0	90.4	91.5	92.8
10 × 10	11.7	13.4	75.4	86.7	87.0	88.7	12.6	14.4	77.3	88.9	89.2	91.2
15 × 15	9.8	11.3	72.7	84.5	85.1	86.8	10.8	12.8	75.4	86.3	87.4	88.6
20 × 20	7.5	9.6	70.2	82.1	82.8	84.6	8.5	10.6	72.7	84.4	85.8	86.2

**Table 5:** Segmentation results of proposed and existing methods on Extended Yale B with pixel corruptions.

Corruption Rate	AC (%)						NMI (%)					
	K-Means	PCA	SSC	LRR	NNLRS	*l_p_*S*p*SS	K-Means	PCA	SSC	LRR	NNLRS	*l_p_*S*p*SS
0.05	11.2	13.1	68.8	83.9	86.9	88.3	14.2	17.1	74.3	89.9	90.8	92.3
0.1	7.6	9.4	64.5	78.3	81.4	83.6	11.6	13.4	68.3	87.4	87.6	89.1
0.15	5.8	8.8	62.4	72.2	76.2	79.2	7.8	9.8	65.4	82.3	83.9	84.5
0.2	3.5	5.6	60.7	68.4	72.8	75.2	4.5	6.6	62.7	77.4	78.8	79.2
